# Measuring Orbital Angular Momentum (OAM) States of Vortex Beams with Annular Gratings

**DOI:** 10.1038/srep40781

**Published:** 2017-01-17

**Authors:** Shuang Zheng, Jian Wang

**Affiliations:** 1 Wuhan National Laboratory for Optoelectronics, School of Optical and Electronic Information, Huazhong University of Science and Technology, Wuhan 430074, Hubei, China

## Abstract

Measuring orbital angular momentum (OAM) states of vortex beams is of great importance in diverse applications employing OAM-carrying vortex beams. We present a simple and efficient scheme to measure OAM states (i.e. topological charge values) of vortex beams with annular gratings. The magnitude of the topological charge value is determined by the number of dark fringes after diffraction, and the sign of the topological charge value is distinguished by the orientation of the diffraction pattern. We first theoretically study the diffraction patterns using both annular amplitude and phase gratings. The annular phase grating shows almost 10-dB better diffraction efficiency compared to the annular amplitude grating. We then experimentally demonstrate the OAM states measurement of vortex beams using annular phase grating. The scheme works well even for high-order vortex beams with topological charge value as high as ± 25. We also experimentally show the evolution of diffraction patterns when slightly changing the fractional topological charge value of vortex beam from 0.1 to 1.0. In addition, the proposed scheme shows potential large tolerance of beam alignment during the OAM states measurement of vortex beams.

Angular momentum can be divided into spin angular momentum (SAM) and orbital angular momentum (OAM) in paraxial beams, which are related to circular polarization and spatial distribution, respectively. In 1992, Allen and coworkers recognized that helically phased beams comprising an azimuthal phase term exp(*ilφ*), have an OAM of *lħ* per photon, where *l* is topological charge value, *φ* is azimuthal angle, and *ħ* is Plank’s constant *h* divided by 2π^1^. In recent years, optical beams carrying OAM (i.e. OAM modes), also referred to as vortex beams, have attracted more and more attention owing to their distinct characteristics such as the phase singularity at the beam center and the resultant doughnut shape intensity profiles. OAM modes with different topological charge value *l*, are theoretically unbounded and orthogonal to each other. Similar to other mode bases in free space or fiber, OAM modes are another basis with which to represent spatial modes. Different mode bases including OAM modes can be employed in mode-division multiplexing (MDM), which a subset of space-division multiplexing (SDM). Very recently, OAM modes have shown great potential for MDM both in free space and fiber-based optical communications^2^,^3^,^4^,^5^. Vortex beams or OAM modes also provide unique opportunities for manipulation of micro-/nano-particles as it asserts torque in addition to forces related to optical intensity gradient, giving rise to orbital and spin movements beyond trapping. Remarkably, for diverse applications of vortex beams in optical communications^2^,^3^,^4^,^5^, optical tweezers^6^,^7^, optical trapping^8^ and quantum information technology^9^, an accurate measurement of the topological charge values of vortex beams, which includes the magnitude and the sign, is of great importance.

Until now, a wide variety of methods have been proposed to generate optical vortex beams^10^,^11^,^12^,^13^,^14^,^15^. Furthermore, quite a few ways have been presented to measure the topological charge value of vortex beams^16^,^17^,^18^,^19^,^20^,^21^,^22^,^23^,^24^. Generally, the light beams carrying OAM can be detected directly by observing the interference patterns. For instance, the measurement of the topological charge value of an optical vortex beam was reported by analyzing the interference pattern between a vortex beam and its mirror image^16^. A triangular aperture was also used for the measurement of vortex beams, which could be up to *l* = ± 7^17^,^18^. Through the diffraction intensity pattern after an annular aperture, the measurement of the topological charge value of an optical vortex beam was also up to |*l|* = 9^19^. The magnitude measurement of the topological charge values of vortex beams using a robust mode converter was reported^20^. A modified Mach–Zehnder interferometer was demonstrated to measure high-order topological charges of vortex beams^21^. In addition, plasmonic lens and plasmonic photodiodes were also used to detect vortex beams^22^,^23^. Recently the gradually-changing-period gratings were designed to measure the OAM states of light beams, which were optimized to detect the OAM beams more sensitively^24^. These well-established schemes achieved impressive operation performance for OAM detection. Another simple method that only uses a tilted convex lens was proposed to enable the measurement of the topological charge magnitude and sign^25^. Despite its simplicity, the footprint of the titled lens is relatively large. The titled lens might also suffer from the aberration-induced performance degradation for OAM detection. Meanwhile, the titled lens method lacks of switchability, reconfigurability, scalability and integration. In this scenario, more diverse approaches would still be expected to flexibly detect OAM states of vortex beams including high-order ones. A laudable goal would be to develop a simple, switchable, reconfigurable and scalable scheme to enable simultaneous magnitude and sign measurement of OAM states of high-order vortex beams. Annual gratings could be possible candidates. Optical annular structures have been used in many other fields, e.g. the generation of cylindrical vector beams^26^,^27^. To the best of our knowledge, measuring OAM states of vortex beams with annular gratings has not yet been reported so far.

In this paper, we propose and demonstrate OAM states measurement of vortex beams using polarization-independent annular gratings. Both annular amplitude and phase gratings are considered for comparison in the theoretical analyses. The annular amplitude grating is simple, while the annular phase grating is efficient. In the experimental works, annular phase grating with better diffraction efficiency is employed. OAM states measurements of high-order vortex beams and fractional vortex beams are all demonstrated in the experiment.

## Concept and principle

**Figure 1:**
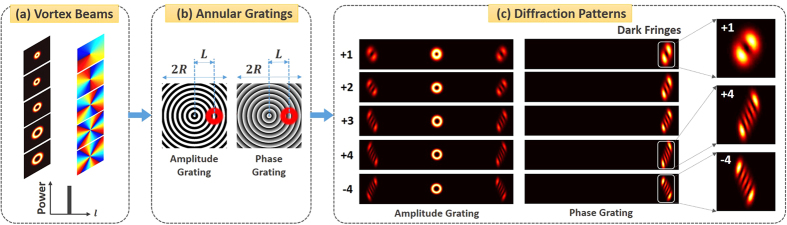
Concept and principle of measuring OAM states of vortex beams with annular gratings. *L*: center distance between annular gratings and vortex beams; R: radius of annular gratings. (**a**) Vortex beams; (**b**) annular gratings; (**c**) diffraction patterns.

The concept and principle of measuring OAM states of vortex beams with annular gratings are illustrated in Fig. 1. The annular gratings can be amplitude grating or phase grating. The vortex beams with different topological charge values (i.e. OAM states), as shown in Fig. 1(a), illuminate the annular gratings at an offset position from the center of the annular gratings (red ring in Fig. 1(b)). The far-field diffraction intensity patterns are recorded after passing through the annular gratings. The magnitude of the topological charge value can be distinguished by observing the number of dark fringes after diffraction. Moreover, the sign of the topological charge value can be determined by observing the orientation of the diffraction pattern. Note that the orientation of the diffraction pattern varies when changing the illumination point on the annular gratings. However, the sign of the topological charge value of vortex beam can still be discriminated as the orientations of diffraction patterns are always different for positive and negative vortex beams. As a consequence, the complete magnitude and sign information of the topological charge value of vortex beams can be measured.

For annular amplitude grating, the transmission function of the annular grating can be expressed as follows


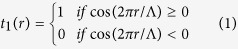


where the transmission of the annular amplitude grating changes along the radial direction *r* with a period of Λ. The annular amplitude grating described by Eq. (1) has a double-sided diffraction, as shown in Fig. 1(c).

To measure the OAM states of vortex beams more efficiently, annular phase grating can be designed to diffract the light beams to single side, as shown in Fig. 1(c). The annular phase grating can be described as follows


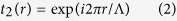


## Simulation Results

**Figure 2:**
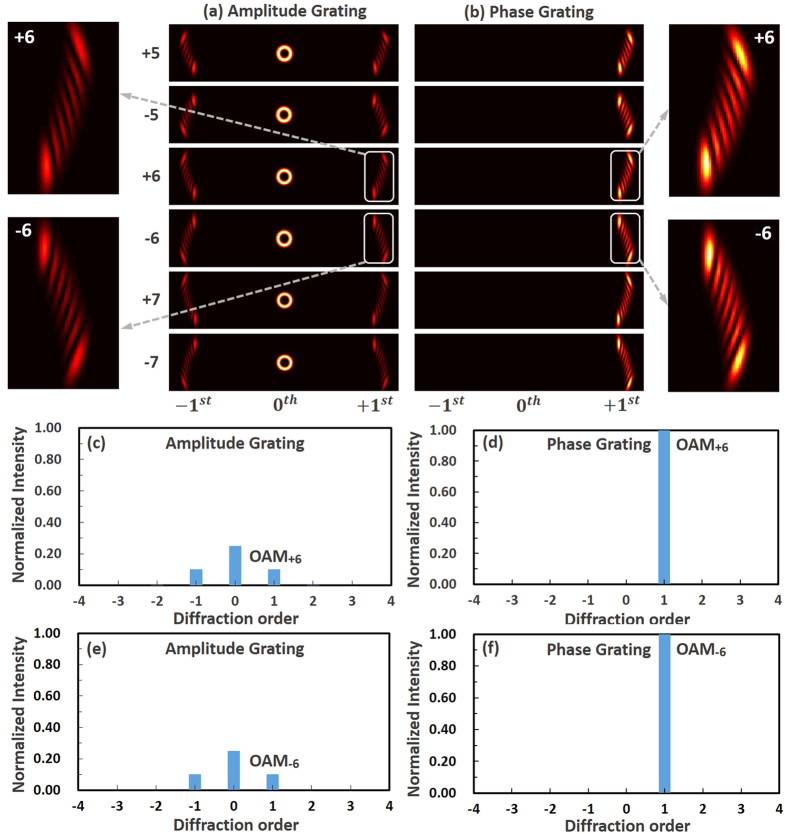
Simulation results of OAM states (*l* = ±5, ±6, ±7) measurement of vortex beams with annular gratings. (**a,c,e**) Annular amplitude grating; (**b,d,f**) annular phase grating; (**a,b**) diffraction patterns; (**c**–**f**) normalized intensity (diffraction efficiency) versus diffraction order; (**c,d**) OAM_+6_; (**e,f**) OAM_−6_.

Based on the fast Fourier transform algorithm, we first theoretically study the diffraction patterns using both annular amplitude and phase gratings. The incident vortex beams are chosen to have topological charge values of *l* = ± 5, ±6, ±7 as typical examples. The beam size of the fundamental Gaussian beam (*l* = 0) is set to be 2 mm, and the relative beam size of vortex beam with topological charge value *l* is 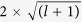 mm. The period of annular gratings is 0.08 mm, and the center distance *L* between annular gratings and incident vortex beams is 5 mm. Figure 2(a) and (b) show simulation results using annular amplitude grating described by Eq. (1) and annular phase grating described by Eq. (2), respectively. One can clearly see that positive and negative vortex beams have distinguishable diffraction patterns with different orientations. Meanwhile, the magnitude of topological charge values can be deduced from the number of dark fringes of diffraction patterns. Although both annular amplitude grating and annular phase grating can be used to measure the OAM states of vortex beams, the operation performance is different. Annular amplitude grating produces zero-order and two first-order diffraction patterns, while annular phase grating only has one first-order diffraction pattern. Figure 2(c–f) depict measured normalized intensity (i.e. diffraction efficiency) as a function of diffraction order for annular amplitude and phase gratings. For annular amplitude grating, the normalized intensity of zero-order and first-order diffraction patterns is ~0.25 and ~0.10, respectively. In contrast, the annular phase grating can diffract almost 100% power of incident light to the positive first order. Thus the annular phase grating offers almost 10-dB better diffraction efficiency compared to the annular amplitude grating.

## Experimental setup

**Figure 3:**
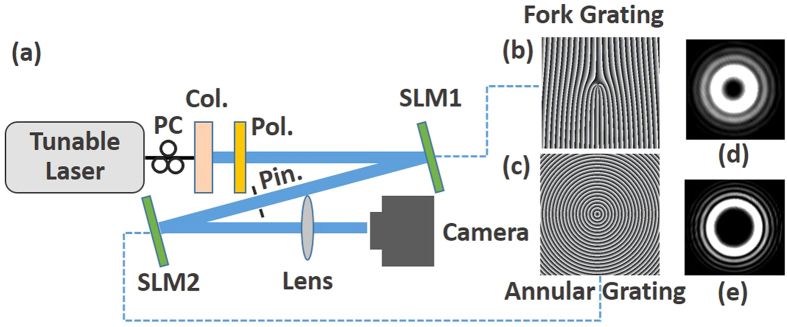
(**a**) Experimental setup; (**b**) fork phase grating loaded to SLM1; (**c**) annular phase grating loaded to SLM2; (**d**) intensity profile of OAM_+2_; (**e**) intensity profile of OAM_+8_.

We then experimentally study the feasibility of the proposed scheme. The experimental setup for measuring the OAM states of vortex beams is shown in Fig. 3(a). In the experiment, two spatial light modulators (SLMs) are employed. SLM1 loaded with fork phase patterns (Fig. 3(b)) is used to generate high-quality vortex beams. SLM2 loaded with annular phase grating (Fig. 3(c)) is used to demonstrate the OAM states measurement of vortex beams. A tunable laser emitting at 1550 nm is coupled to free space and collimated by a collimator (Col.) with a beam size of ~2.5 mm. A linear polarizer (Pol.) aligned to the working direction of SLM1 is used to ensure linearly polarized Gaussian beam projected onto SLM1. A polarization controller (PC) before the Col. is used to maximize the optical power of incident light. After the generation of vortex beams when reflecting off the SLM1, annular phase grating loaded onto the SLM2 is used to measure the OAM states of vortex beams. A pinhole (Pin.) inserted between SLM1 and SLM2 is used to select the high-quality vortex beams and block other unwanted orders during the generation of vortex beams by SLM1 with fork phase patterns. A lens is placed after the SLM2 and the far-field diffraction pattern is observed by a camera. Considering the Gaussian beam size and the effective area of the SLMs (15.36 × 8.64 mm^2^) in the experiment, the period of the annular phase grating is chosen to be 0.12 mm, and the center distance *L* between annular phase grating and incident vortex beams is about 7.7 mm. Figure 3(d) and (e) show measured intensity profiles of two examples of generated vortex beams with topological charge values of *l* = + 2 and *l* = + 8.

**Figure 4:**
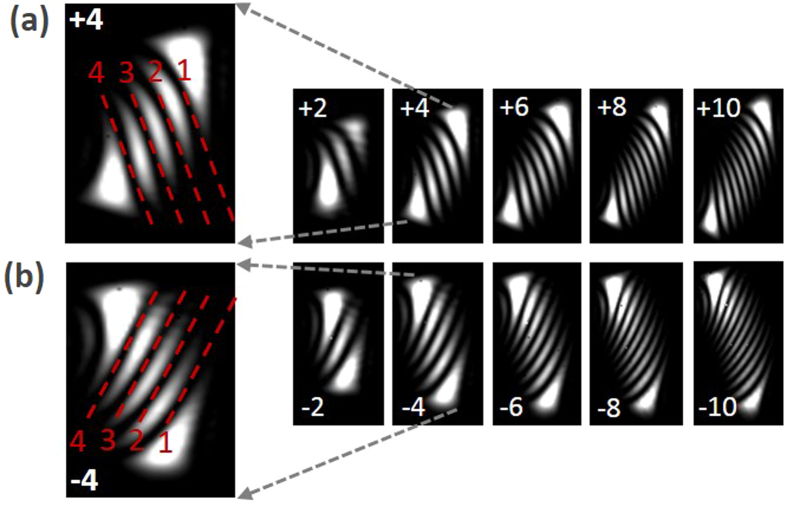
Measured diffraction patterns of vortex beams with *l* = ±2, ±4, ±6, ±8, ±10.

Figure 4 shows experimental results for OAM states measurement of vortex beams with topological charge values of *l* = +2, +4, +6, +8, +10 and *l* = −2, −4, −6, −8, −10. The obtained experimental results agree well with theoretical analyses shown in Fig. 2. The first row in Fig. 4 shows the OAM states measurement of positive vortex beams with *l* = +2, +4, +6, +8, +10, and the second row is the OAM states measurement of negative vortex beams with *l* = −2, −4, −6, −8, −10. The inset Fig. 4(a) and (b) depict enlarged OAM states measurement of vortex beams with *l* = + 2 and *l* = −2. The number of red dashed lines representing dark fringes indicates the magnitude of the topological charge value of incident vortex beam. The orientations of two diffraction patterns of vortex beams *l* = + 2 and *l* = −2 are different, determining the sign of the topological charge value of incident vortex beam.

**Figure 5:**
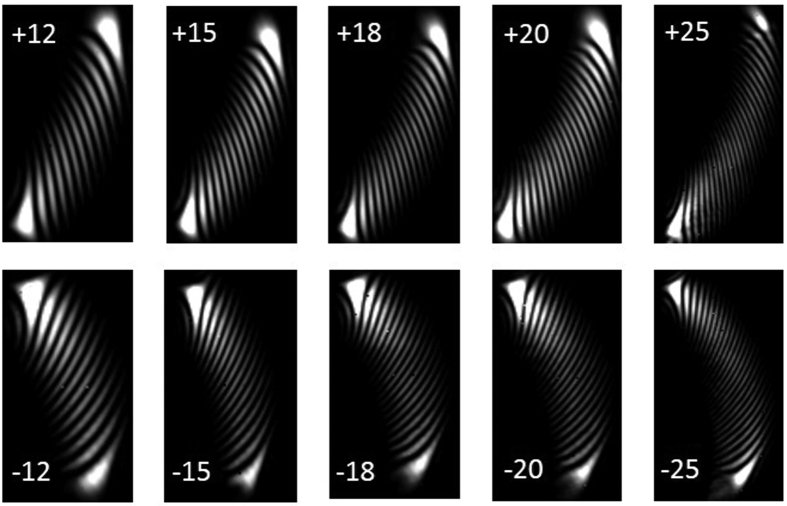
Measured diffraction patterns of vortex beams with *l* = ±12, ±15, ±18, ±20, ±25.

To further verify the scalability of the proposed scheme to measure high-order vortex beams. We show the measured diffraction patterns of vortex beams with *l* = ±12, ±15, ±18, ±20, ±25. As shown in Fig. 5, the number of dark fringes increases with the order of vortex beam. Actually, the number of dark fringes is always equal to the order of vortex beam (i.e. magnitude of topological charge value of vortex beam). In particular, one can still observe countable 25 dark fringes even for high-order vortex beams with *l* = ± 25. Moreover, the orientations of diffraction patterns of positive and negative vortex beams are easy to be distinguished. Thus the proposed scheme works well even for high-order vortex beams with topological charge value as high as ± 25.

**Figure 6:**
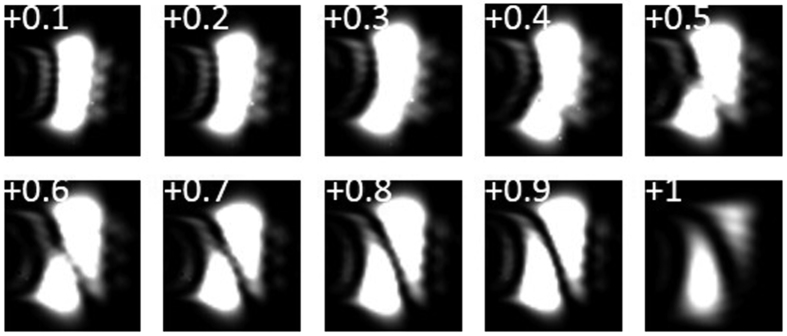
Measured diffraction patterns of vortex beams with fractional topological charge value *l* = 0.1~1.0.

We also measure the diffraction patterns of vortex beams with fractional topological charge value. As shown in Fig. 6, we record the evolution of diffraction patterns when slightly changing the topological charge value of vortex beam from 0.1 to 1.0 with a varying spacing of 0.1. One can see a bright spot diffraction pattern with *l* close to 0, gradual splitting of diffraction pattern with *l* = 0.1~0.9, and one clear dark fringe with *l* = 1.0.

**Figure 7:**
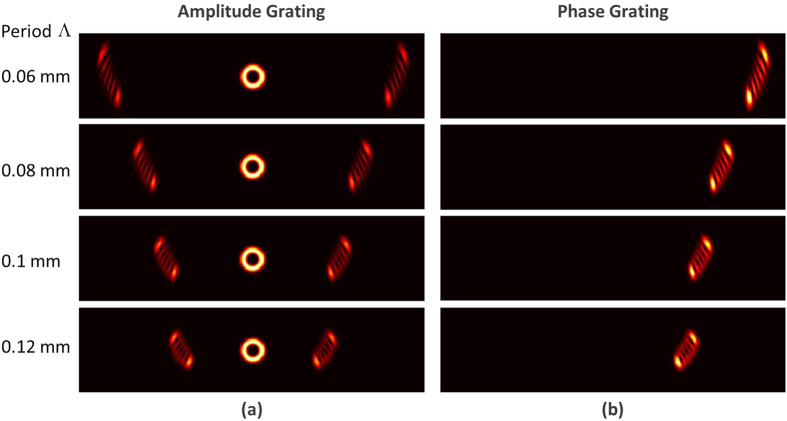
Simulation results of OAM states (*l* = +5) measurement of vortex beams with annular gratings. (**a**) Amplitude grating with period Λ = 0.06 mm, 008 mm, 0.1 mm and 0.12 mm; (**b**) phase grating with period Λ = 0.06 mm, 008 mm, 0.1 mm and 0.12 mm.

**Figure 8:**
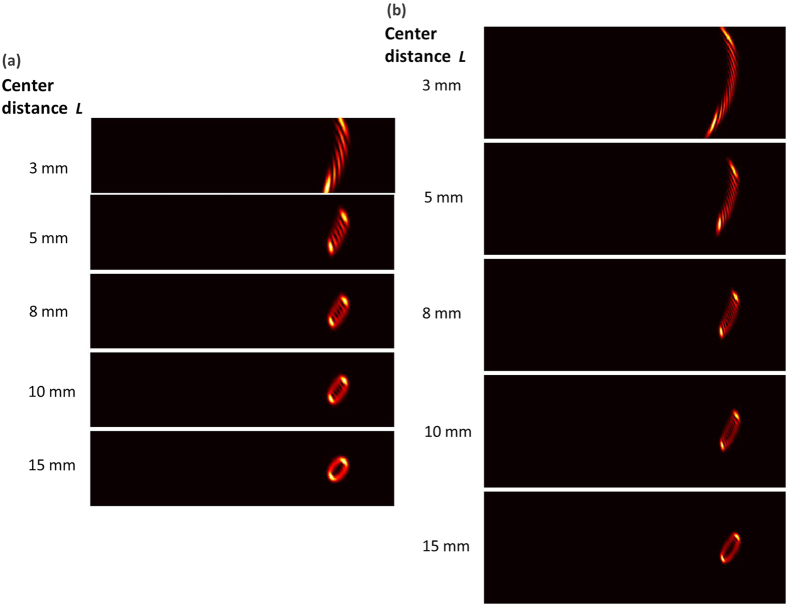
Simulation results of OAM states (*l* = +5, +10) measurement of vortex beams with annular phase gratings. (**a**) The diffraction patterns of vortex beams (*l* = + 5) with center distance between annular gratings and incident vortex beams *L* = 3 mm, 5 mm, 8 mm, 10 mm and 15 mm; (**b**) the diffraction patterns of vortex beams (*l* =+ 10) with center distance between annular gratings and incident vortex beams *L* = 3 mm, 5 mm, 8 mm, 10 mm and 15 mm.

**Figure 9:**
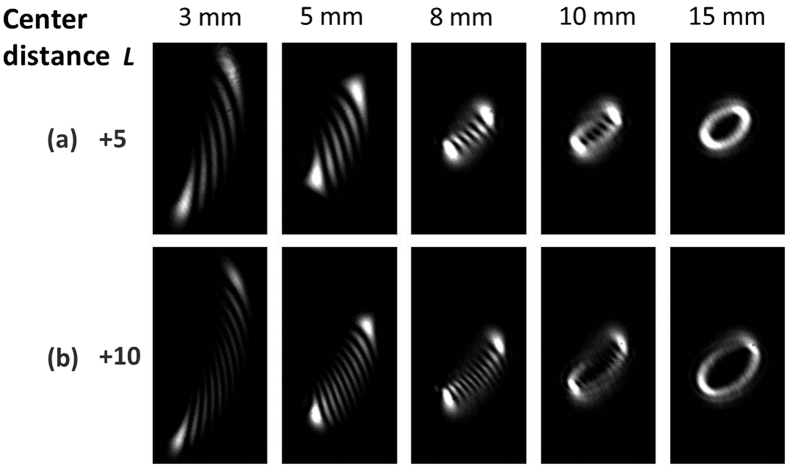
Experimental results of OAM states (*l* = +5, +10) measurement of vortex beams with annular phase gratings. (**a**) Measured diffraction patterns of vortex beams (*l* = +5) with different center distances between annular gratings and incident vortex beams *L* = 3 mm, 5 mm, 8 mm, 10 mm and 15 mm; (**b**) measured diffraction patterns of vortex beams (*l* = +10) with different center distances between annular gratings and incident vortex beams *L* = 3 mm, 5 mm, 8 mm, 10 mm and 15 mm.

To verify the reliability of the proposed scheme, we further study the influence of structure parameters (the period of annular gratings Λ and the center distance *L* between annular gratings and incident vortex beams) on the diffraction patterns. When the period of annular gratings varies, the diffraction angle of diffraction patterns also changes accordingly. Moreover, when the period of annular gratings is much smaller than the size of incident vortex beams, the diffraction patterns become clear and easily identified, as shown in Fig. 7. Limited by the resolution of the employed spatial light modulator loading annular gratings, the period of annular gratings is chosen to be 0.12 mm in the experiment which is smaller enough compared to the size of incident vortex beams to ensure clear diffraction patterns. As shown in Fig. 8, when the center distance *L* between the annular phase grating and incident vortex beams becomes larger, the incident vortex beams feel reduced effect of the curvature grating, resulting in degraded diffraction patterns. Actually, the annular grating functions almost as a conventional uniform grating as the center distance *L* is relatively large. The evolution process of the diffraction patterns with the increase of center distance *L* is clearly shown in Fig. 8. We also demonstrate the impact of the center distance *L* on the diffraction patterns in the experiment, as shown in Fig. 9. One can see distinguishable diffraction patterns when the center distance *L* increases from 3 to 8 mm. Further increase of the center distance *L* degrades the quality of the diffraction patterns.

Additionally, the polarization independence is important for practical applications. It is noted that the annular gratings are polarization independent, therefore, the proposed scheme to measure the OAM states of vortex beams with annular gratings is also polarization independent.

## Discussion

In conclusion, we propose and demonstrate a simple and efficient method to measure OAM states (topological charge values) of vortex beams by exploiting annular gratings. The magnitude and sign of topological charge values are determined by the number of dark fringes and the orientation of the diffraction patterns, respectively. Using fast Fourier transform algorithm, we theoretically analyze the diffraction patterns with both annular amplitude and phase gratings. 10-dB better diffraction efficiency is achievable when employing annular phase grating. Using commercially available SLM loaded with annular phase grating in the experiment, we demonstrate OAM states measurement of vortex beams even with high-order topological charge values as high as ± 25 and fractional topological charge values from 0.1 to 1.0.

The presented OAM measurement method based on annular gratings shows several distinct features as follows.

1. The annular grating method has relatively large tolerance of beam alignment during the OAM states measurement of vortex beams.
2. The footprint of the annular grating structure can be relatively small. It might be free of the aberration-induced performance degradation for OAM detection compared with the lens method.
3. The annular grating method based on SLM is switchable and reconfigurable. It is possible to flexibly adjust the OAM detection result simply by changing the loaded the annular grating onto the SLM.
4. The annular grating method is also scalable for robust measurement of different orders of OAM, multiple OAM states and even an OAM array.
5. With future improvement, the annular grating could be also fabricated with a ultra-small volume assisted by photonic integration such as nanophotonic annular grating on silicon platforms. It is expected that the photonic integrated annular grating has nano-scale high resolution and could be much more compact.

## Methods

For both annular amplitude and phase gratings, the far-field diffraction intensity distribution of the incident vortex beam after passing through the annular gratings can be written by^20^


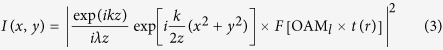


where OAM_*l*_ denotes the field of the OAM-carrying vortex beam, 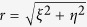, (*x, y*) is the coordinate of the far-field, (ξ, *η*) is the coordinate of the annular gratings, *l* is the topological charge value of the OAM-carrying vortex beam, *k* is the wavenumber and *F* represents the Fourier Transform. In the simulation, we choose Laguerre-Gaussian beam as the OAM-carrying vortex beam. The field of the incident Laguerre-Gaussian beam can be expressed as


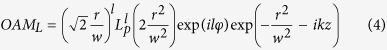


where *w* is the waist size of the fundamental mode, 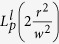 is the associated Laguerre polynomial, *p* is the radial topological charge (*p* = 0 is assumed), *l* is the topological charge and *φ* is the angle.

## Additional Information

**How to cite this article**: Zheng, S. and Wang, J. Measuring Orbital Angular Momentum (OAM) States of Vortex Beams with Annular Gratings. *Sci. Rep.* **7**, 40781; doi: 10.1038/srep40781 (2017).

**Publisher's note:** Springer Nature remains neutral with regard to jurisdictional claims in published maps and institutional affiliations.
